# Elevated ABCB1 Expression Confers Acquired Resistance to Aurora Kinase Inhibitor GSK-1070916 in Cancer Cells

**DOI:** 10.3389/fphar.2020.615824

**Published:** 2021-01-14

**Authors:** Zhuo-Xun Wu, Yuqi Yang, Jing-Quan Wang, Wen-Min Zhou, Junyu Chen, Yi-Ge Fu, Ketankumar Patel, Zhe-Sheng Chen, Jian-Ye Zhang

**Affiliations:** ^1^ Department of Pharmaceutical Sciences, College of Pharmacy and Health Sciences, St. John’s University, Queens, NY, United States; ^2^ Key Laboratory of Molecular Target & Clinical Pharmacology and the State Key Laboratory of Respiratory Disease, School of Pharmaceutical Sciences & The Fifth Affiliated Hospital, Guangzhou Medical University, Guangzhou, China

**Keywords:** multidrug resistance, GSK-1070916, aurora kinase inhibitor, ATP-binding cassette transporter, ABCB1, substrate

## Abstract

The emergence of multidrug resistance (MDR) has been a major issue for effective cancer chemotherapy as well as targeted therapy. One prominent factor that causes MDR is the overexpression of ABCB1 transporter. In the present study, we revealed that the Aurora kinase inhibitor GSK-1070916 is a substrate of ABCB1. GSK-1070916 is a newly developed inhibitor that is currently under clinical investigation. The cytotoxicity assay showed that overexpression of ABCB1 significantly hindered the anticancer effect of GSK-1070916 and the drug resistance can be abolished by the addition of an ABCB1 inhibitor. GSK-1070916 concentration-dependently stimulated ABCB1 ATPase activity. The HPLC drug accumulation assay suggested that the ABCB1-overexpressing cells had lower levels of intracellular GSK-1070916 compared with the parental cells. GSK-1070916 also showed high binding affinity to ABCB1 substrate-binding site in the computational docking analysis. In conclusion, our study provides strong evidence that ABCB1 can confer resistance to GSK-1070916, which should be taken into consideration in clinical setting.

## Introduction

The emergence of multidrug resistance (MDR) has been a major concern to cancer therapy. MDR is characterized as the development of insensitivity of cancer cells to an array of anticancer drugs with different structures and mechanisms of action (Bukowski et al., 2020). The acquired drug resistance may occur in multiple stages, occurring initially through rare cell variability in transcription, and later developing into stable epigenetic reprogramming (Robak et al., 2018). MDR is a multifactorial phenomenon that involves comprehensive mechanisms, including mutation of oncogenes, enhanced DNA damage repair, overexpression of drug efflux pumps (Wang et al., 2019). The dysregulation of oncogenes as well as tumor suppressor genes are often associated with the constitutive activation of phosphorylation. Inhibiting kinase phosphorylation may represent an effective strategy for cancer treatment. For instance, selective Pin1 inhibitors can inhibit the phosphorylation of Pin1, thereby inhibit the downstream signal pathways and disrupt the proliferation of cancer cells (Wu et al., 2019a). However, the mutations or alterations in the targeted oncogene, such as KRAS and EGFR, can impair the response to therapy (Lohr et al., 2014). DNA repair is another well-established MDR mechanism that can confer cancer cells resistance to chemotherapeutic agents such as platinum-based agents and DNA topoisomerase inhibitors (Mansoori et al., 2017). Among all the mechanisms that lead to MDR, one prominent factor contributing to MDR is the overexpression of ATP-Binding Cassette (ABC) transporters. These ABC transporters function as efflux pumps to extrude its substrates from the cells. Therefore, they serve a protective role in maintaining normal physiological function but also cause MDR in cancer cells.

ABCB1 (also known as MDR1 or P-glycoprotein) was the first ABC transporter to be identified by Ling et al. and is by far the most studied MDR-related efflux pump (Juliano and Ling, 1976). ABCB1 is constitutively expressed in organs and physiological barriers, including kidney, intestine, and the blood-brain barrier (Gonçalves et al., 2020). It has been extensively reported that overexpression of ABCB1 in cancer cells can confer resistance to chemotherapeutic agents such as paclitaxel, vincristine, colchicine, as well as tyrosine kinase inhibitors (TKIs) such as imatinib and dasatinib (Dohse et al., 2006; Hegedus et al., 2009). In recent decades, the focus of ABCB1 in cancer research has gradually shifted to the development of a therapeutic strategy to overcome ABCB1-mediated MDR. Combining ABCB1 inhibitors with ABCB1 substrate drugs may re-establish the drug sensitivity in drug-resistant cells, thereby enhancing therapeutic effect in cancer patients (Li et al., 2016). Given the significant role that ABCB1 plays in pharmacokinetics and pharmacodynamics, the FDA suggests that all new drug candidates be screened for potential interaction with ABCB1 transporter (Crawford et al., 2018).

GSK-1070916 is a novel, potent TKI targeting the Aurora B/C kinases (Adams et al., 2010). Aurora B kinase actively involves in cytokinesis and chromosome segregation during mitosis (Kallio et al., 2002; Hauf et al., 2003). The function of Aurora C kinase is not well established, but studies suggested it has similar function to Aurora B kinase (Sasai et al., 2004; Yan et al., 2005). There are several Aurora kinase inhibitors that have progressed to clinical development, including tozasertib (Zhang et al., 2020), barasertib (Floc’h et al., 2019) and MK-5108 (Amin et al., 2016). Previous studies showed that GSK-1070916 has a broad antitumor effect on cancer cell lines and in tumor xenograft models including lung, breast, colorectal, and leukemia (Hardwicke et al., 2009). Other studies showed that GSK-1070916 has potential effects against T-cell acute lymphoblastic leukemia (Spartà et al., 2014). The drug has a synergistic effect in combination with EGFR/ERBB inhibitor neratinib in MYC-amplified cells (Uitdehaag et al., 2015). To date, the phase I trial investigating the dosage and toxicity of GSK-1070916 has been completed. Given the significant antitumor effect and the progress achieved by other Aurora kinase inhibitors, GSK-1070916 may deserve further investigation in clinical trials. However, barasertib was suggested to be a substrate of ABCB1 (Marchetti et al., 2013). CCT129202 also showed interaction with ABCB1 and reversed ABCB1-mediated MDR (Cheng et al., 2012). As there is no available data regarding the interaction of GSK-1070916 and the ABCB1 transporter, investigating such interaction may provide insight for the design of future experiments and clinical trials.

In this study, we demonstrated that overexpression of ABCB1 can render cancer cells resistant to GSK-1070916. Because the inclusion of an ABCB1 inhibitor can minimize the drug resistance, the combination of GSK-1070916 with ABCB1 inhibitor may provide additional benefit to cancer patients with high tumor ABCB1 expression.

## Materials and Methods

### Chemicals

GSK-1070916 was a gift from ChemieTek (Indianapolis, IN). GSK-1070916 and chemotherapeutic drugs were dissolved in DMSO at a stock concentration of 10 mM. All other chemicals were obtained from Sigma Chemical Co (St. Louis, MO) unless listed otherwise.

### Cell Lines and Cell Culture

The following cell lines were cultured as previously described (Wu et al., 2019b). Briefly, KB-3-1 and its colchicine-selected ABCB1-overexpressing subline KB-C2, SW620 and its doxorubicin-selected ABCB1-overexpressing subline SW620/Ad300, HEK293/pcDNA3.1 and HEK293/ABCB1 cells were cultured in DMEM with 10% FBS. KB-C2 cells were maintained in the presence of colchicine (2 μg/ml), SW620/Ad300 cells were maintained in the presence of doxorubicin (0.3 μg/ml). Transfected cell lines were maintained in the presence of 2 mg/ml G418. All cell lines were maintained in a humid incubator (37°C, 5% CO_2_) and subcultured at 80% confluency.

### Cytotoxicity Evaluation

MTT assay was applied to determine the cytotoxicity of anticancer drugs. Briefly, cells were seeded into 96-well plates (5 × 10^3^/well). Following cell attachment, the cells were treated with different concentrations of GSK-1070916 or chemotherapeutic drugs, in the presence or absence of verapamil for 72 h. After additionally incubated with MTT solution for 4 h, DMSO was added to dissolve the formazan crystals. The plates were read at 570 nm by microplate reader to obtain the optical density values.

### Evaluation of ABCB1 ATPase Activity

The ABCB1-associated ATPase activity was measured in the presence of GSK-1070916 (0–40 μM) as previously described (Wu et al., 2020b). Briefly, the ABCB1 ATPase activity was determined using ATPase assay kit from BD Biosciences (San Jose, CA). Insect cell membranes (20 μg) were incubated in an assay buffer. Then, GSK-1070916 (0–40 µM) was incubated with the membrane vesicles for 3 min. The ATP hydrolysis was initialized by adding 5 mM of Mg-ATP and terminated by adding 5% SDS. The inorganic phosphate was quantified with a spectrophotometer at 880 nm.

### HPLC GSK-1070916 Accumulation Assay

The HPLC assay was carried out as previous described (Wu et al., 2020a). Cells were seeded into 6-well plates (2 × 10^5^ cell/well) and incubated for 48 h before assay. At the day of treatment, plain medium (DMEM without FBS) with 20 μM of GSK-1070916 with or without 5 μM of verapamil was added into designated wells and incubated for another 2 h. Thereafter, cells were harvested and centrifuged at 14,000 rpm for 10 min. The supernatant was subjected to HPLC analysis.

### [^3^H]-Paclitaxel Accumulation Assay

Cells were seeded into 24-well plates (1 × 10^5^ cells/well). At the following day, cells were pretreated with or without GSK-1070916 or verapamil for 2 h at 37°C. Thereafter, cells were incubated in the medium with 10 nM of [^3^H]-paclitaxel (Moravek Biochemicals Inc, Brea, CA) in the presence or absence of GSK-1070916 or verapamil for another 2 h. Finally, cells were transferred into scintillation vials and the radioactivity of samples were measured by a Packard TRICARB 1900CA liquid scintillation analyzer (Packard Instrument, Downers Grove, IL).

### Immunoblotting

After cells were incubated with GSK-1070916 for 72 h, the lysates were collected. The protein concentration of protein samples was measured by BCA assay kit (Thermo Scientific, Rockford, IL). The immunoblots were performed as previously described (Wu et al., 2020c). Briefly, the samples were subjected to SDS-PAGE, then transferred onto PVDF membranes. The membranes were incubated with 5% skim milk for 2 h, then immunoblotted with anti-ABCB1 or anti-GAPDH antibodies (Thermo Fisher Scientific Inc., Waltham, MA) overnight at 4°C, followed by 2 h immunoblotting with anti-mouse secondary antibody (Cell Signaling Technology Inc., Danvers, MA) at room temperature. The immunoreactive bands were detected with ECL reagents (Thermo Fisher Scientific Inc., Waltham, MA). ImageJ software (NIH, MD) was used to measure the densitometry of immunoreactive bands.

### Immunofluorescence Assay

Cells were seeded into 24-well plates at the density of 2 × 10^5^ cells per well. The cells were then treated with 3 μM of GSK-1070916 for up to 72 h. After incubation, the cells were fixed, permeabilized and blocked in PBS with 6% BSA (VWR chemicals, LLC, Radnor, PA). Thereafter, the cells were incubated with anti-ABCB1 antibody (Thermo Fisher Scientific Inc., Waltham, MA) overnight at 4°C. Then, cells were further incubated with Alexa Fluor 488 conjugated anti-mouse secondary antibody (Thermo Fisher Scientific Inc., Waltham, MA) for 2 h at room temperature. The nuclei were stained with DAPI solution. The immunofluorescence image was visualized using a Nikon TE-2000S fluorescence microscope (Nikon Instruments Inc., Melville, NY).

### *In Silico* Molecular Docking Analysis

The GSK-1070916 structure was constructed for docking analysis with human ABCB1 model (6QEX) (Alam et al., 2019). Docking calculations were performed in AutoDock Vina (version 1.1.2) (Trott and Olson, 2010). Hydrogen atoms and partial charges were added using AutoDockTools (ADT, version 1.5.4). Docking grid center coordinates were determined from the bound ligand mitoxantrone provided in 6QEX PDB files. The top-scoring pose (sorted by affinity score: kcal/mol) was selected for further analysis and visualization.

### Statistical Analysis

The experiments were repeated three times and data are expressed as mean ± standard deviation (SD). The data were analyzed using GraphPad software (Prism 8.1). Statistical analysis was performed through one-way ANOVA. In all cases, *p* value less than 0.05 between each group was considered significant.

## Results

### The Antiproliferative Effect of GSK-1070916 in Parental and ABCB1-Overexpressing Cells

**FIGURE 1:**
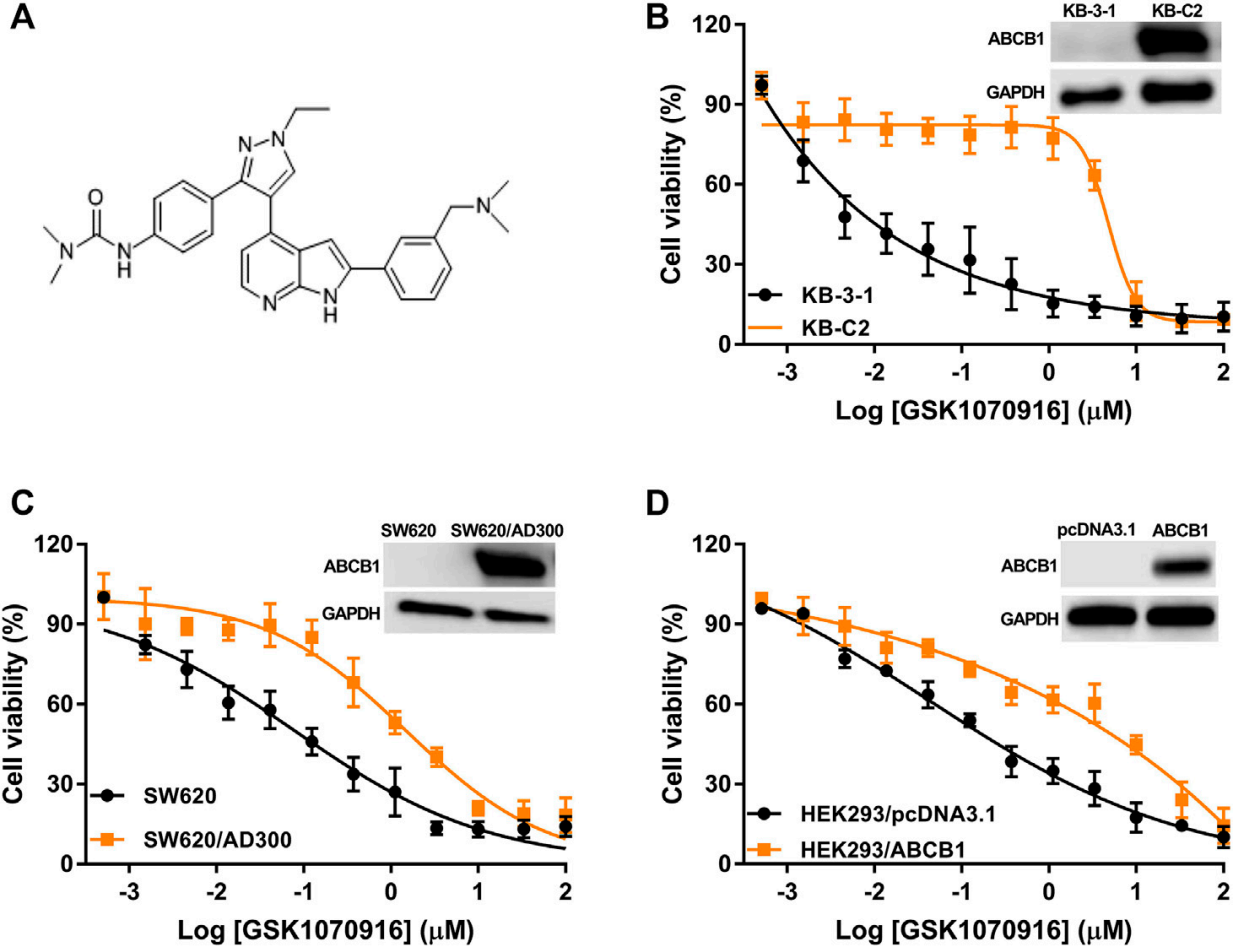
The cytotoxicity of GSK-1070916 in parental and ABCB1-overexpressing cell lines. **(A)** Chemical structure of GSK-1070916; cell viability curves for **(B)** KB-3-1 and KB-C2 cells; **(C)** SW620 and SW620/Ad300 cells; **(D)** HEK293/pcDNA3.1 and HEK293/ABCB1 cells. Data are expressed as mean ± SD from a representative of three independent experiments (n = 3).

The chemical structure of GSK-1070916 is presented in Figure 1A. To evaluate the potential interaction of GSK-1070916 and ABCB1, three pairs of cell lines were selected to examine the cytotoxicity profile. Western blot was performed to confirm the overexpression of ABCB1 in drug-selected KB-C2, SW620/Ad300 and gene-transfected HEK293/ABCB1 cells (Figure 1B–D). All three ABCB1-overexpressing cells were resistant to GSK-1070916 as compared with the corresponding parental cells. The colchicine-selected KB-C2 cells and the doxorubicin-selected SW620/Ad300 cells showed 83.3- and 15.28-fold resistance to GSK-1070916, respectively. In addition, ABCB1 gene-transfected HEK293 cells also showed 7.66-fold resistance to GSK-1070916 as compared to the parental HEK293/pcDNA3.1 cells. The IC_50_ values and resistance fold were calculated and presented in Table 1.

**TABLE 1:** The cytotoxicity of GSK-1070916 in cells overexpressing the ABCB1 transporter.

Treatment	IC_50_ value ± SD^a^ (μM, Resistance fold^b^)	
	GSK-1070916	GSK-1070916 + verapamil 5 μM
KB-3-1	0.016 ± 0.003 (1.00)	0.015 ± 0.005 (0.91)
KB-C2	1.359 ± 0.253 (83.30)^c^	0.212 ± 0.057 (13.00)^c^
SW620	0.096 ± 0.054 (1.00)	0.106 ± 0.070 (1.11)
SW620/AD300	1.460 ± 0.546 (15.28)^c^	0.212 ± 0.098 (2.22)
HEK293/pcDNA3.1	0.435 ± 0.041 (1.00)	0.585 ± 0.050 (1.34)
HEK293/ABCB1	3.331 ± 0.816 (7.66)^c^	0.646 ± 0.029 (1.48)

^a^ IC_50_ values are represented as mean ± SD of at least three independent experiments.

^b^ Rf: Resistance fold was calculated by dividing the IC_50_ values of substrates in the presence or absence of inhibitor by the IC_50_ of parental cells without inhibitor.

^c^ p < 0.05 vs. the parental control group without reversal agent.

### ABCB1 Inhibitor Restored the Sensitivity of ABCB1-Overexpressing Cells to GSK-1070916

In order to confirm that overexpression of ABCB1 can confer resistance to GSK-1070916, reversal studies were carried out. The combination of ABCB1 inhibitor verapamil with GSK-1070916 was able to significantly antagonize drug resistance. In the presence of 5 μM verapamil, GSK-1070916 resistance decreased from 83.3-fold to 13-fold and from 15.28-fold to 2.22-fold in KB-C2 and SW620/Ad300 cells, respectively. A similar trend was observed in HEK293/ABCB1 cells, with the resistance fold decreasing from 7.66-fold to 1.48-fold, suggesting a complete reversal of GSK-1070916 resistance. As shown in Supplementary Figure S1, the combination of GSK-1070916 and verapamil showed synergistic effect in ABCB1-overexpressing KB-C2 cells. In contrast, the combination treatment showed no significant effect in parental KB-3-1 cells, suggesting that verapamil may increase the cytotoxicity of GSK-1070916 by antagonizing ABCB1-mediated MDR. Therefore, the results support the hypothesis that overexpression of ABCB1 is a prominent factor leading to GSK-1070916 resistance.

### GSK-1070916 Significantly Stimulated ABCB1 ATPase Activity

**FIGURE 2:**
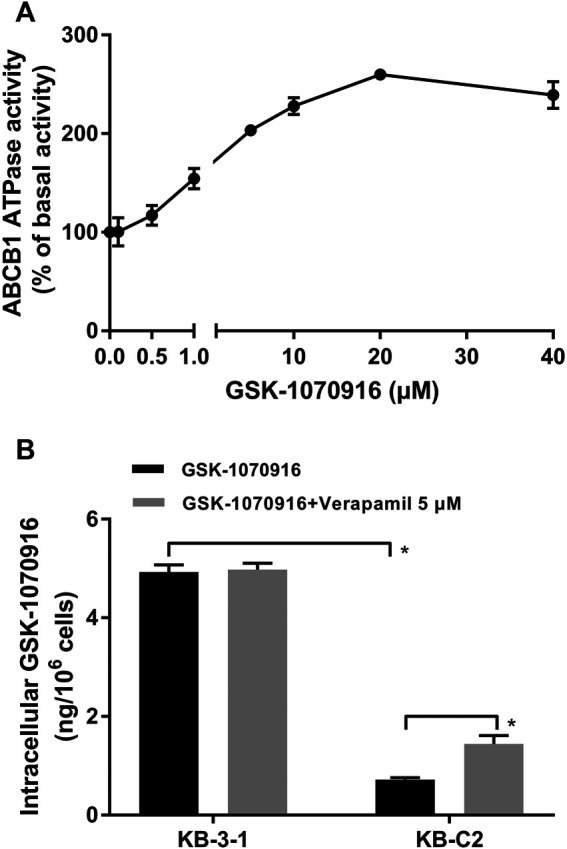
GSK-1070916 stimulated ABCB1 ATPase activity and was transported out of ABCB1-overexpressing cells. **(A)** The effect of GSK-1070916 on ABCB1 ATPase activity at concentration range from 0 to 40 μM. **(B)** The intracellular accumulation of GSK-1070916 in KB-3-1 and KB-C2 determined by HPLC assay. Data are expressed as mean ± SD derived from three independent experiments (n = 3). *p < 0.05 vs. the control group.

It is suggested that, in ABCB1 transporter, ATP hydrolysis is a crucial step to provide energy for substrate efflux activity. To further confirm that GSK-1070916 is an ABCB1 substrate, ATPase assay was performed to measure the ABCB1-related ATP hydrolysis in membrane vesicles incubated with GSK-1070916 (0–40 μM). If a drug can stimulate the ATPase activity, it suggests that the drug can interact with the transporter at the substrate-binding site. As presented in Figure 2A, GSK-1070916 showed concentration-dependent stimulatory effect on the ABCB1 ATPase activity. The stimulatory effect of GSK-1070916 reached 50% maximal effect (EC50) at 0.83 μM and to a maximum of 2.6-fold of basal activity, suggesting that GSK-1070916 can interact with ABCB1 at the substrate-binding site.

### Intracellular Accumulation of GSK-1070916 in Parental and ABCB1-Overexpressing Cells

Since our finding suggested that GSK-1070916 is a transported substrate of ABCB1, HPLC assay was carried out to validate if ABCB1-overexpressing cells can pump out GSK-1070916. As shown in Figure 2B, the intracellular accumulation of GSK-1070916 was 7-times lower in drug-resistant KB-C2 cells compared to parental KB-3-1 cells. Co-treatment with ABCB1 inhibitor, verapamil, was able to partially increase the drug accumulation in drug-resistant cells without affecting that in the parental cells. Therefore, the HPLC assay provided another direct evidence to show that overexpression of ABCB1 can lead to GSK-1070916 resistance.

### GSK-1070916 Partially Reversed ABCB1-Mediated Multidrug Resistance

**FIGURE 3:**
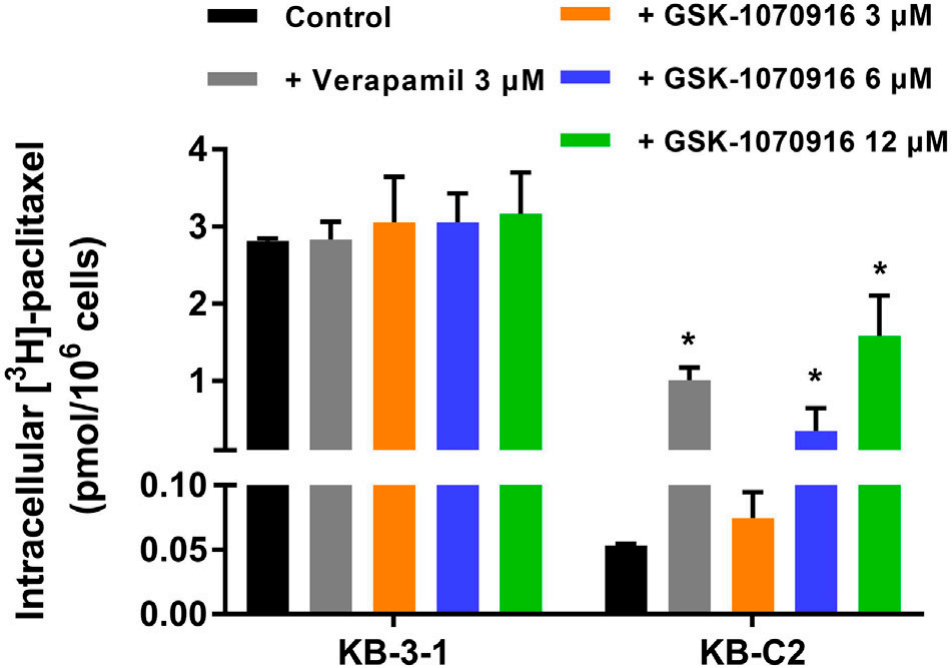
Effect of GSK-1070916 on the accumulation of [^3^H]-paclitaxel. The intracellular accumulation of [^3^H]-paclitaxel in KB-3-1 and KB-C2 cells after incubation with 3, 6, 12 μM of GSK-1070916 for 2 h. Data are expressed as mean ± SD derived from three independent experiments (n = 3). *p < 0.05 vs. the control group.

To explore the relationship between GSK-1070916 and ABCB1, [^3^H]-paclitaxel accumulation assay was performed. Verapamil was used as a positive ABCB1 reversal agent in this assay. Although the concentration used in this assay was significantly toxic in the MTT assay, the short time treatment prevented GSK-1070916 from affecting normal cellular function. As shown in Figure 3, there was a 55-fold difference between the control group of parental KB-3-1 cells and the ABCB1-overexpressing KB-C2 cells with over 90% of the paclitaxel being pumped out from the KB-C2 cells. In addition, co-treatment with verapamil was able to enhance the retention of paclitaxel in KB-C2 cells, suggesting an active efflux function of ABCB1. At high concentrations, GSK-1070916 showed the ability to increase the accumulation of [^3^H]-paclitaxel. At 12 μM, GSK-1070916 significantly hindered ABCB1 efflux activity, with less than 50% of paclitaxel being pumped out from the KB-C2 cells. In contrast, 3 μM of GSK-1070916 demonstrated no effect to the accumulation of [^3^H]-paclitaxel in ABCB1-overexpressing KB-C2 cells. Notably, none of the treatment altered paclitaxel accumulation in parental cells. These results suggested that GSK-1070916, at high concentrations, can compete with other substrates for the ABCB1 transporter.

### GSK-1070916 did Not Alter ABCB1 Protein Expression Level or Cell Membrane Localization

**FIGURE 4:**
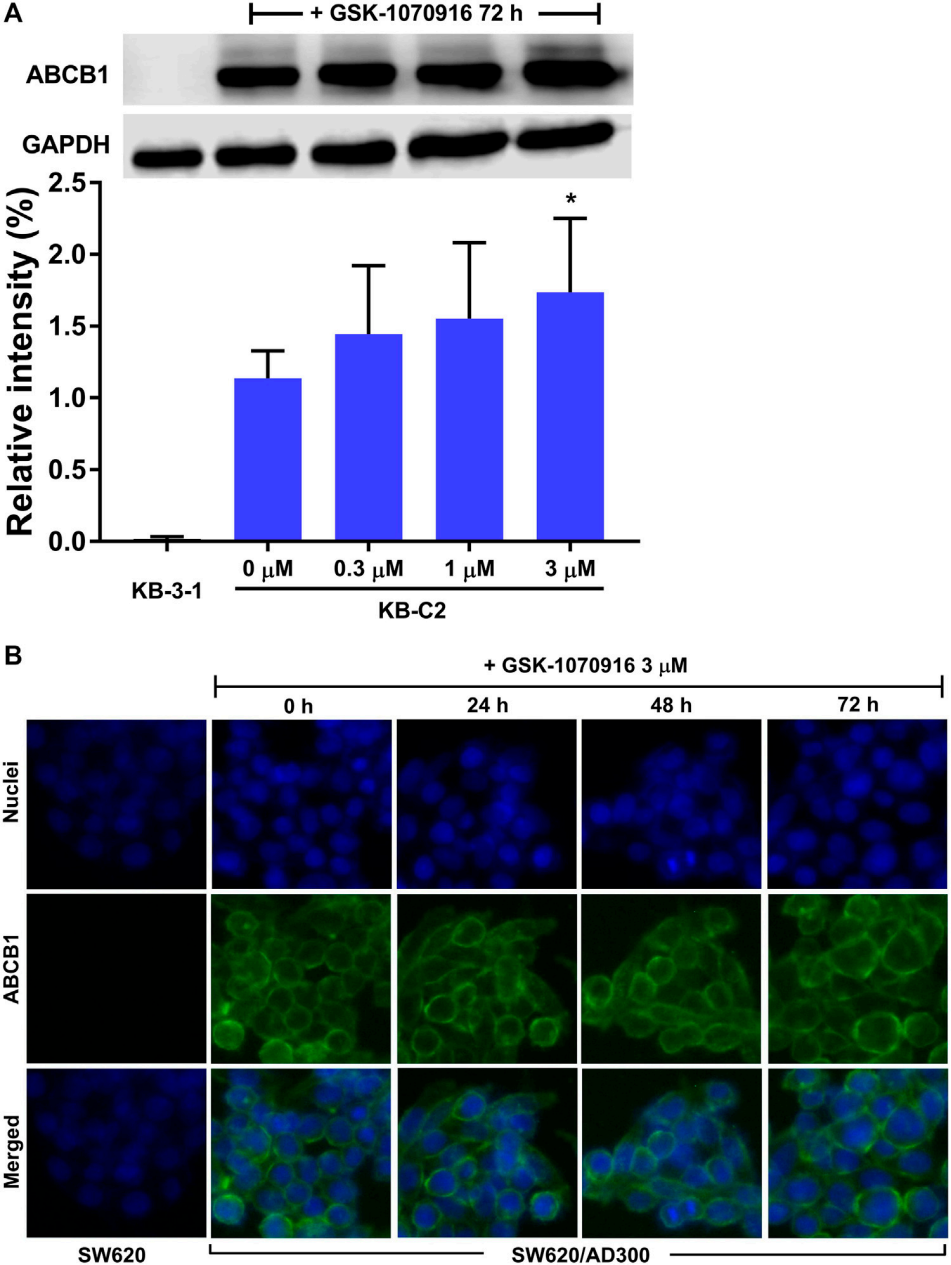
The effect of GSK-1070916 on the ABCB1 protein expression level and membrane localization. **(A)** The effect of 0–3 μM of GSK-1070916 on the expression level of ABCB1 in KB-C2 cells after 72 h treatment. **(B)** Cell membrane localization of ABCB1 in SW620/Ad300 cells incubated with 3 μM of GSK-1070916 for 72 h. Data are expressed as mean ± SD derived from three independent experiments (n = 3). *p < 0.05 vs. the control group.

Substrate drugs can sometimes upregulate the protein expression of ABCB1, which may enhance the drug resistance. Considering this possibility, Western blot was performed to evaluate the short-term effect of GSK-1070916 on the ABCB1 expression level. As shown in Figure 4A, the nontoxic concentration of GSK-1070916 demonstrated no significant effect to the protein expression of ABCB1 for up to 72 h treatment, compared to the control group. Another possible interaction between GSK-1070916 and ABCB1 is to alter the membrane localization of ABCB1. Consequently, we performed immunofluorescence assay to visualize the localization of ABCB1 in ABCB1-overexpressing SW620/Ad300 cells. As presented in Figure 4B, the localization of ABCB1 transporter is indicated by the green fluorescence. The incubation of SW620/Ad300 cells with 3 μM of GSK-1070916 for up to 72 h did not alter the cell membrane localization of ABCB1 as compared with the untreated group (Figure 4B).

### Docking Analysis

**FIGURE 5:**
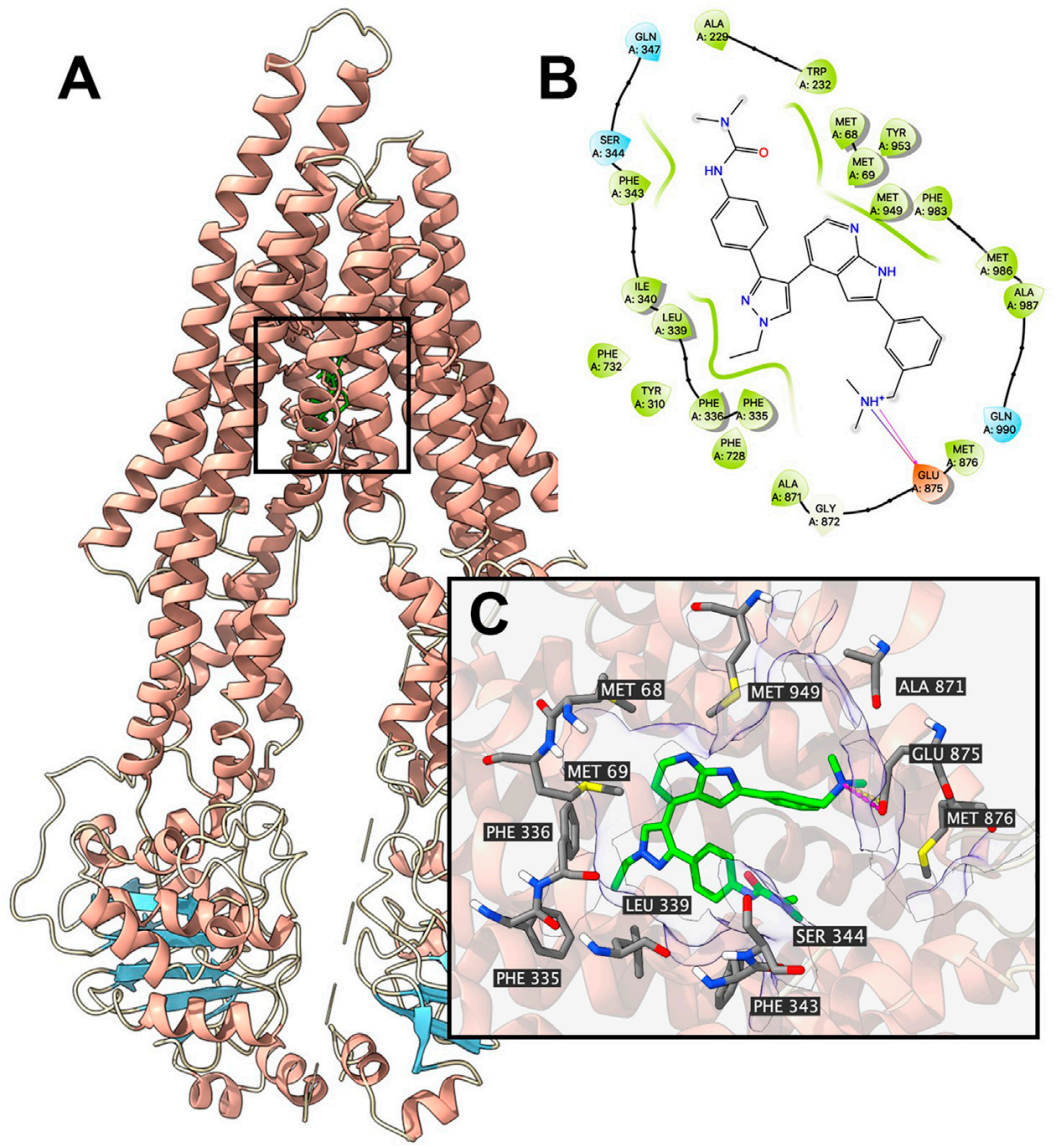
Interaction between GSK-1070916 and human ABCB1 protein model. Interaction between GSK-1070916 and human ABCB1 protein. **(A)** Overview of the best-scoring pose of GSK-1070916 in the drug binding pocket of ABCB1 protein (6QEX). ABCB1 was displayed as colored ribbons (helix: red; strand: blue; coil: white). GSK-1070916 is displayed as colored sticks. Carbon: lime; oxygen: red; nitrogen: blue, hydrogen: white. **(B)** 2D diagram of the interaction between GSK-1070916 and ABCB1 binding pocket. Important amino acids within 3 Å from the ligand are displayed as colored bubbles (green: hydrophobic; blue: polar). Purple solid lines with arrow indicate hydrogen bonds. Purple solid lines without arrow indicate halogen bonds. **(C)** Details of the interaction between GSK-1070916 and ABCB1 (6QEX) binding pocket. ABCB1 helices are displayed as colored ribbons (helix: red; strand: blue; coil: white). Important residues are displayed as colored sticks (carbon: gray; oxygen: red; nitrogen: blue; hydrogen: white). GSK-1070916 is displayed as colored sticks (same as in A). Hydrogen bonds are displayed as magenta dash lines. Halogen interactions are displayed as yellow dash lines. Molecular surface formed by displayed residues is shown as purple solid planes.

Since the ATPase assay showed that GSK-1070916 has a stimulatory effect toward ABCB1 ATPase, docking simulation was performed in the substrate-binding site (6QEX) of ABCB1. Our results showed that GSK-1070916 can bind to the substrate-binding site with an affinity score of −8 kcal/mol. Figure 5 depicted the detailed interaction between GSK-1070916 and ABCB1. GSK-1070916 is positioned in the hydrophobic cavity formed by Met68, Met69, Trp232, Ser344, Phe343, Phe336, Leu339, Ala871, Met876, Ala987 and Met986. Additionally, the amide group in GSK-1070916 was stabilized by a hydrogen bond and a halogen bond formed with Glu875.

## Discussion

In recent decades, Aurora kinases have become attractive targets for cancer treatment. The Aurora kinases can be divided into Aurora-A, -B and -C based on their functionality and localization. These kinases play a crucial role in cell proliferation by regulating the mitotic phase of the cell cycle (Borisa and Bhatt, 2017). To date, there are several Aurora kinase inhibitors, such as alisertib, tozasertib, barasertib and danusertib, undergoing phase 2 or phase 3 clinical trials (Falchook et al., 2015). Optimal therapeutic effect was demonstrated in these clinical trials, therefore highlighting the potential role of Aurora kinase inhibitors in cancer treatment. GSK-1070916 is a new Aurora kinase inhibitor which showed promising anticancer effects in pre-clinical studies (Hardwicke et al., 2009). Given the potent anticancer effect of the drug, GSK-1070916 is being progressed to clinical trials. However, one major issue for cancer therapy is the emergence of MDR. Particularly, it has been well established that the overexpression of ABCB1 transporter mediates MDR in cancer cells (Lage, 2016). ABCB1 serves as a membrane efflux pump that extrude its substrates from the cancer cells, thereby interfering with the therapeutic effect of a diverse range of anticancer drugs (Goldstein et al., 1990; Mahon et al., 2003). Despite the difference in chemical structure, several Aurora kinase inhibitors were identified as substrate of ABCB1, such as barasertib (Marchetti et al., 2013), VX-680 and ZM447439 (Tavanti et al., 2013). Hence, it is vital to explore the potential interaction of ABCB1 and GSK-1070916 and propose rational combination strategies to overcome drug resistance.

In this study, we revealed that overexpression of ABCB1 can render cancer cells resistant to GSK-1070916, which may challenge its therapeutic effect in clinical setting. We used two ABCB1-overexpressing cancer cell lines, KB-C2 and SW620/Ad300. Both cell lines are highly resistant to paclitaxel, colchicine and doxorubicin (Ji et al., 2018). In addition, HEK293 cells transfected with *ABCB1* gene was included in this study. Since drug-selected cancer cells may develop MDR due to multiple mechanisms, the drug resistance mechanism in HEK293/ABCB1 is only via overexpression of ABCB1 transporter. Firstly, the antiproliferative effect of GSK-1070916 was examined in different cell lines. Both drug-selected and gene-transfected ABCB1-overexpressing cells showed significant resistance to GSK-1070916 compared to the corresponding parental cells. In addition, the inclusion of ABCB1 inhibitor verapamil was able to restore the sensitivity of ABCB1-overexpressing cells to GSK-1070916. This trend is similar to other ABCB1 substrate drugs such as imatinib (Maia et al., 2018), eribulin (Oba et al., 2016), WYE-354 (Wang et al., 2020), which the anticancer efficacies are attenuated in ABCB1-overexpressing cells and the drug resistance can be abolished by ABCB1 inhibitors. Hence, we hypothesized that GSK-1070916 may be a substrate of ABCB1. Furthermore, ABCB1-related ATPase activity in the presence of GSK-1070916 was measured. It is generally proposed that when a substrate binds to the substrate-binding site in transmembrane domains (TMDs), it promotes conformational changes in nucleotide-binding domains (NBDs). The binding and hydrolysis of ATP in NBDs cause conformational changes in the TMDs, resulting in the translocation of substrate to the extracellular space (Yakusheva and Titov, 2018). It is suggested that the ATPase activity of ABCB1 depends on the concentration of the transported substrates (Yamaguchi, 2016). In the ATPase assay, GSK-1070916 concentration-dependently stimulated the activity of ABCB1 ATPase. The maximum stimulation of GSK-1070916 is approximately 2.5-fold, which is comparable to other reported substrates such as volasertib (3-fold, (Wu et al., 2015)), ricolinostat (1.5-fold, (Wu et al., 2018)), WYE-354 (1.4-fold, (Wang et al., 2020)), suggesting GSK-1070916 is a transported substrate of ABCB1. In addition, the computational docking analysis also indicated that GSK-1070916 has a high binding affinity to ABCB1 substrate-binding site.

Our hypothesis was further validated in the HPLC drug accumulation assay. Since drug resistance is a complicated phenomenon with multiple possible mechanisms, the drug resistance to GSK-1070916 can be achieved by several ways, such as pumping the drug into extracellular matrix or metabolizing the drug into inactive form. Therefore, direct measurement of intracellular drug accumulation provides a strong evidence to identify if the drug is a transported substrate of ABCB1. After 2 h incubation with GSK-1070916, a significant difference of drug accumulation was observed between parental cells and ABCB1-overexpressing cells. The co-treatment with ABCB1 inhibitor verapamil was able to increase the accumulation of GSK-1070916 in ABCB1-overexpressing cells but not in the parental cells, confirming that ABCB1 is the main factor contributing to decreased drug accumulation in these cells. Combining this result with the cytotoxicity profile, it suggested that ABCB1 can pump the drug out of cancer cells, resulting in decreased sensitivity of cancer cells to GSK-1070916. After identifying GSK-1070916 as a substrate of ABCB1, we investigated whether it can act as am inhibitor and reverse ABCB1-mediated MDR by occupying the ABCB1 transporter as seen in other MDR reversal studies (Levy et al., 2019). The [^3^H]-paclitaxel accumulation assay was introduced to explore this possibility. Paclitaxel is a well-known substrate of ABCB1, and therefore GSK-1070916 was hypothesized to compete with paclitaxel for the substrate-binding site, leading to increased intracellular accumulation of paclitaxel. Our results showed that GSK-1070916 can concentration-dependently increase the intracellular accumulation of paclitaxel in drug-resistant cells without affecting that in the parental cells. However, high concentrations were required to achieve the reversal effect, and therefore this may weaken its role as a rational ABCB1 inhibitor. It should be noted that the concentrations used in this experiment is higher than the IC_50_ and can be toxic to the cancer cells. However, the 4 h incubation of GSK-1070916 with cancer cells may not affect to the cellular function. This is confirmed in the parental cells that no difference was observed between the vehicle group and the treatment groups. Subsequently, we investigated whether GSK-1070916 can affect the protein expression and/or cell membrane localization of ABCB1. Within 72 h treatment, GSK-1070916 demonstrated no effect on ABCB1 expression level or membrane localization. However, whether long-term treatment with GSK-1070916 can stimulate ABCB1 expression remain inconclusive and should be further explored.

The computational docking analysis is an efficient tool to predict the interaction of ligands with proteins (Rosano et al., 2013; Lionta et al., 2014). The method has widely applied in the field of biology and pharmacology (Kontoyianni, 2017). Although the computational analysis does not reveal the actual binding interaction of ligands with proteins, it has become a reliable method in screening substrates and reversal agents of ABC transporters (Ferreira et al., 2017). The docking analysis indicated that GSK-1070916 interact with the drug-binding site of ABCB1 (−8 kcal/mol), which is comparable to other ABCB1 substrates such as WYE-354 and ricolinostat (Wu et al., 2018; Wang et al., 2020).

In conclusion, our study provides strong evidence to demonstrate that overexpression of ABCB1 is sufficient to confer cancer cells resistant to GSK-1070916. Therefore, ABCB1-mediated MDR should be taken into consideration when investigating the anticancer effect of GSK-1070916 in the clinical setting. Future study may focus on the potential interaction of GSK-1070916 with other MDR-linked ABC transporters.

## Data Availability Statement

The original contributions presented in the study are included in the article/Supplementary Material, further inquiries can be directed to the corresponding authors.

## Author Contributions

Conceptualization, Z-XW and Z-SC; methodology, Z-XW, YY, J-QW, Y-GF, KP, and Z-SC; writing-original draft preparation, W-MZ and Z-XW; writing-review and editing, Z-XW, J-YZ, and Z-SC; supervision, J-YZ and Z-SC. All authors have read and agreed to the published version of the manuscript.

## Funding

This study was supported partially by National Natural Science Foundation grant (81872901 and U1903126), Guangdong Basic and Applied Basic Research Foundation (2020A1515010605).

## Conflict of Interest

The authors declare that the research was conducted in the absence of any commercial or financial relationships that could be construed as a potential conflict of interest.
