# Supplementary material for: Elevated ABCB1 Expression Confers Acquired Resistance to Aurora Kinase Inhibitor GSK-1070916 in Cancer Cells
Source: Front Pharmacol. 2021 Jan 14;11:615824. doi: 10.3389/fphar.2020.615824 (PMC7841342; doi:10.3389/fphar.2020.615824)
Supplement: Supplementary file 1 [file image1.pdf]

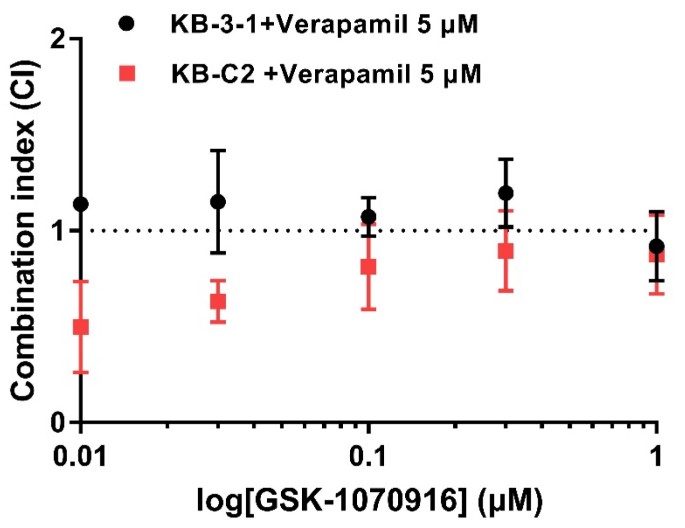

Figure S1. The combination index of GSK-1070916 with verapamil in KB-3-1 and KB-C2 cells. GSK-1070916 plus verapamil was synergic only in ABCB1-overexpressing KB-C2 cells but not in parental KB-3-1 cells. Data are expressed as mean ± SD derived from three independent experiments (n=3).
